# Emergence of Invasive Serotype Ib Sequence Type 10 Group B Streptococcus Disease in Chinese Infants Is Driven by a Tetracycline-Sensitive Clone

**DOI:** 10.3389/fcimb.2021.642455

**Published:** 2021-05-14

**Authors:** Li Zhang, Wen-Juan Kang, Lei Zhu, Li-Jun Xu, Chao Guo, Xin-Hua Zhang, Qing-Hua Liu, Lan Ma

**Affiliations:** ^1^ Department of Clinical Laboratory, Shanxi Children’s Hospital Shanxi Maternal and Child Health Hospital, Taiyuan, China; ^2^ Department of Neonatology Department, Shanxi Children’s Hospital Shanxi Maternal and Child Health Hospital, Taiyuan, China; ^3^ Department of Pathophysiology, Shanxi Medical University, Taiyuan, China

**Keywords:** group B streptococcus, infants, late-onset disease, tetracycline, meningitis

## Abstract

**Background:**

Group B streptococcus (GBS) is a leading cause of serious infections in infants. The extensive use of tetracycline has led to the selection of specific resistant and infectious GBS clones. The sequence type (ST) 10 GBS strain, causing invasive infections in infants, is becoming prevalent in China. We aimed to understand the clinical and microbiological characteristics of this GBS strain.

**Methods:**

We conducted a retrospective study on infants with invasive GBS disease from the largest women’s and children’s medical center in Shanxi and collected data between January 2017 and October 2020. GBS isolates were analyzed by capsule serotyping, genotyping, antibiotic resistance, and surface protein genes.

**Results:**

All ST10 isolates belonged to serotype Ib; type Ib/ST10 strains were responsible for 66.7% (14/21, *P* < 0.05) of infant invasive GBS infections during the period and all resulted in late-onset (LOD) and late LOD disease (14/14). Infants with type Ib/ST10 GBS disease had significantly higher rates of meningitis (9/14, 64.3%, p < 0.05) and clinical complications (5/14, 35.7%, p < 0.05). The Ib/ST10 GBS isolates had limited genetic diversity, clustered in the CC10/bca/PI-1 + PI-2a genetic lineage, showed resistance to erythromycin, lincomycin, and fluoroquinolones and sensitivity to tetracycline, and possessed genes *ermT*, *ermB*, and amino acid changes in *gyrA* and *parC*.

**Conclusions:**

The probable clonal expansion can result in severe infections in infants and ongoing emergence of multi-drug resistant isolates. Continued monitoring for type Ib/ST10 GBS infections is warranted.

## Introduction

Group B streptococcus (GBS) is a leading cause of serious infection in young infants, commonly resulting in sepsis, pneumonia, focal infections, bacteremia, and meningitis, which leads to a serious death rate (Lawn et al., 2017; Madrid et al., 2017). GBS infections can be further divided based on onset: early onset (EOD; birth to 7 days), late-onset (LOD; 8 to 90 days), and late LOD disease (LLOD; older than 3 months of age). The current gold standard for preventing the transfer of GBS from mother to neonate in many countries is the use of *intrapartum* antibiotic prophylaxis during delivery (Lawn et al., 2017; Li et al., 2017; Furfaro et al., 2018). Although this precaution has reduced the incidence of GBS-associated EOD, the rates of LOD with GBS infections remain unchanged.

Conventionally, descriptive GBS epidemiology is based on capsular serotyping or genotyping (Lawn et al., 2017; Madrid et al., 2017; Furfaro et al., 2018). Ten serotypes (Ia, Ib, and II-IX) have been identified according to their capsular polysaccharide antigens, and five of them (Ia, Ib, II, III, and V) occupied approximately 96% of all neonatal invasive GBS infections globally. Multilocus sequence typing (MLST) has been used to target seven conserved housekeeping genes in classified GBS strains into numerous sequence types (STs) and clonal complexes (CCs), of which ST1, ST10, ST17, ST19, and ST23 are more frequently associated with invasive infection. Furthermore, clustered regularly interspaced short palindromic repeats (CRISPRs) have been recently identified in most archaea and many bacteria (Gholizadeh et al., 2017; Koonin and Makarova, 2019; Westra et al., 2019). This technique is a novel and effective way to reliably research population-wide GBS genomics (Sorek et al., 2008; Hidalgo-Cantabrana et al., 2018).

The general role of pathogens as the world’s leading cause of neonatal disease is a concern for antibiotic resistance in GBS strains and the selective pressures we have imposed through extensive use of antibiotic drugs, such as tetracycline (Da Cunha et al., 2014; Lawn et al., 2017; Madrid et al., 2017). Penicillin is a medication of choice for prophylaxis and GBS disease therapy, with erythromycin and clindamycin recommended for patients with a *ß*-lactam allergy. However, second-line resistance to antibiotics continues to be strong, and the production of new phenotypes in GBS populations is observed. Even worse, reports of multi-drug resistant isolates are becoming more prevalent (Campisi et al., 2016; Guo D. et al., 2018; Hayes et al., 2020).

III/ST17 GBS strains have been particularly well studied given their role as the major cause of severe, invasive infant disease (Tazi et al., 2010; Furfaro et al., 2018; Ratner, 2019; Tazi et al., 2019). We recently reported a high incidence of isolation for both carriage and invasive ST10 GBS in Shanxi (Zhang et al., 2020), Northern China. Ji et al. also described that the percentage of invasive GBS cases caused by serotype Ib (93.4% were ST10) was higher in both the Northern and Northeastern regions of China compared with others (Ji et al., 2019). Despite the clear and growing impact of ST10 GBS strains, there remains limited data concerning the clinical and microbiological properties of this GBS strain.

Here, we aimed to (i) describe the recent ST10 manifestations and outcomes of infants with invasive GBS disease, and (ii) investigate the microbiological characteristics of the ST10 GBS strain by determining its capsule serotyping, CRISPR profiles, antibiotic resistance, and surface protein genes.

## Materials and Methods

The study protocol was authorized by the ethics committee of Shanxi Children’s Hospital Shanxi Maternal and Child Health Hospital. Neither study-specific nor any other measures are needed for this study, and the data were analyzed in anonymity. Accordingly, the ethics committee waived the provision for written informed consent by registered patients.

### Study Design and Strain Collection

We isolated GBS strains (n = 21) from infants with invasive GBS disease from January 2017 to October 2020. These cases were found retrospectively from the database of Shanxi Children’s Hospital Shanxi Maternal and Child Health Hospital, a tertiary teaching hospital that houses 1,600 beds.

We classified invasive GBS disease sepsis, meningitis, pneumonia, or focal infections based on tests and diagnoses of laboratories reported in the medical registers of the patients. Sepsis was characterized as the bacterial increase of GBS in the blood drawn from the peripheral vein and with symptoms associated with sepsis. Meningitis was defined as the presence of clinical symptoms with positive results on cerebrospinal fluid (CSF) culture or sepsis with CSF pleocytosis in patients with a negative CSF culture. Pneumonia was defined as the presence of clinical symptoms with radiological findings and positive results on sputum or bronchoalveolar lavage fluid culture. Focal infection disease was defined as the presence of clinical symptoms and positive results for pyogenic fluids and tissue culture. Diseases between 0 and 7 days and 8 to 90 days respectively were described as EOD and LOD. The disease was listed as LLOD after 90 days of age (Lawn et al., 2017; Madrid et al., 2017).

The corresponding medical professionals undertook a retrospective chart analysis. We collected the following: demographic data and information on the clinical and laboratory observations, presence of meningitis, antimicrobial susceptibility of the pathogen, and clinical outcomes at discharge.

### Capsule Serotyping and MLST

A multiplex PCR assay for identifying serotypes Ia to IX of GBS developed by Imperi et al. (2010) was conducted. The PCR is amplified and sequenced to seven housekeeping genes (adhP, pheS, atr, glnA, sdhA, glcK, and tkt) (Jones et al., 2003). The allele, ST and CC research was conducted in the MLST database (https://pubmlst.org/organisms/streptococcus-agalactiae) and a goeBURST program.

### Antimicrobial Susceptibility

Susceptibility to penicillin G, vancomycin, erythromycin, clindamycin, levofloxacin, cefepime, ceftriaxone, cefotaxime, tetracycline, and linezolid was measured by performing Kirby–Bauer disk diffusion according to the Clinical and Laboratory Standards Institute (Clinical and Laboratory Standards Institute, 2020).

### Resistance and Surface Protein Genes

Using conventional PCR amplification, PCR assays were performed using primers and references from **Table 1** (Kong et al., 2002; Creti et al., 2004; Murayama et al., 2009; Madzivhandila et al., 2013; Guo Y. et al., 2018; Nie, 2018). We targeted genes associated with surface protein and antimicrobial resistance. The former included GBS alpha (*bca*) and alpha-like (*eps, rib, alp2, alp3*, and *alp4*) surface protein genes and the pilus islands (PI) (PI-1, PI-2a, and PI-2b). The latter included macrolide and tetracycline resistance genes, *ermB*, *ermA*, *ermT*, *mefA/E*, *tetM*, *tetK*, *tetL*, and *tetO*; lincosamide resistance genes, *lnuB;* fluoroquinolone (FQ) resistance genes encoding DNA gyrase A (*gyrA*) and topoisomerase IV C (*parC*). Sequence comparison analyses were conducted using Vector NTI software (Invitrogen, Grand Island, NY, USA). The reference sequences of *gyrA* and *parC* (SDSE strain YZ1605, GenBank accession number: CP026082.1) were obtained through GenBank.

**Table 1:** The primers used in the resistance and surface protein genes test and their reference.

Genes	Primer	Sequence (5′ to 3′)	Ref./isolate/ID^a^
alpha and alpha-like surface protein genes	Forward	TGATACTTCACAGACGAAACAACG	Creti et al., 2004
	alpha C-R	TACATGTGGTAGTCCATCTTCACC	
	Rib-R	CATACTGAGCTTTTAAATCAGGTGA	
	Epsilon-R	CCAGATACATTTTTTACTAAAGCGG	
	Alp2/3-R	CACTCGGATTACTATAATATTTAGCAC	
	Alp4-R	TTAATTTGCACCGGATTAACACCAC	
	Alp2-F	CAGACTGTTAAAGTGGATGAAGATATTACCTTTACGG	Kong et al., 2002
	Alp2-R	GGTATCTGGTTTATGACCATTTTTCCAGTTATACG	
antimicrobial resistance associated genes	ermA-F	CCCGAAAAATACGCAAAATTTCAT	Nie S. P., 2018
	ermA-R	CCCTGTTTACCCATTTATAAACG	
	ermB-F	TGGTATTCCAAATGCGTAATG	
	ermB-R	CTGTGGTATGGCGGGTAAGT	
	mefA/E-F	CAATATGGGCAGGGCAAG	
	mefA/E-R	AAGCTGTTCCAATGCTACGG	
	tetM-F	GTGGACAAAGGTACAACGAG	
	tetM-R	CGGTAAAGTTCGTCACACAC	
	tetO-F	AACTTAGGCATTCTGGCTCAC	
	tetO-R	TCCCACTGTTCCATATCGTCA	
	tetK-F	GATCAATTGTAGCTTTAGGTGAAGG	
	tetK-R	TTTTGTTGATTTACCAGGTACCATT	
	tetL-F	TGGTGGAATGATAGCCCATT	
	tetL-R	CAGGAATGACAGCACGCTAA	
	lnuB-F	CCTACCTATTGTTTGTGGAA	Guo Y. et al., 2018
	lnuB-R	ATAACGTTACTCTCCTATTC	
	gyrA-F	GGTTTAAAACCTGTTCATCGTCGT	Murayama et al., 2009
	gyrA-R	GCAATACCAGTTGCACCATTGACT	
	parC-F	CCGGATATTCGTGATGGCTT	
	parC-R	TGACTAAAAGATTGGGAAAGGC	
pilus islands	PI-1-F	CTACCAACGGCCAAGCTATTTACC	Madzivhandila et al., 2013
	PI-1-R	TAGCCGCTTTTTCATTCTTTCTCC	
	PI-2a-F	AACTCCCTATATTTGCAGGTTCAA	
	PI-2a-R	CGGGTGTAACGACTTTTATCTGAT	
	PI-2b-F	GGGGGTAGGCTTAATGGCTTAT	
	PI-2b-R	TCCGGTTTAACTGTTCTGATTTGAT	

a Ref, Reference.

### CRISPR Analysis

The CRISPR1 loci were amplified and sequenced as previously described Beauruelle et al., 2017). CRISPR1 regions were amplified by using the oligonucleotide pairs CRISPR1-PCRF/CRISPR1-PCRR. Then PCR products were sequenced by using internal sequencing primers CRISPR-SEQF/CRISPR-SEQR. CRISPR sequences were stored and analyzed by using the CRISPRs web server (https://crispr.i2bc.paris-saclay.fr/). This site acts as a gateway to publicly accessible CRISPRs database and software. It enables the easy detection of CRISPRs in locally produced data and consultation of CRISPRs present in the database. As a result, the CRISPR1 loci were divided into direct repeats and corresponding spacer sequences. Each unique spacer was numbered manually, and the result was analyzed and modified according to Lier et al. (2015).

### Phylogenetic and Statistical Analysis

A neighbor-joining phylogenetic tree with an interior-branch test was constructed using MEGA-X. Analyses were conducted *via* SPSS software, v. 19.0. A Student’s t-test, chi-square test, and Fisher’s exact test were used for comparing proportions when necessary. All statistical testing was two-sided, and P <0.05 was set in significance.

## Results

### Isolates

**Figure 1:**
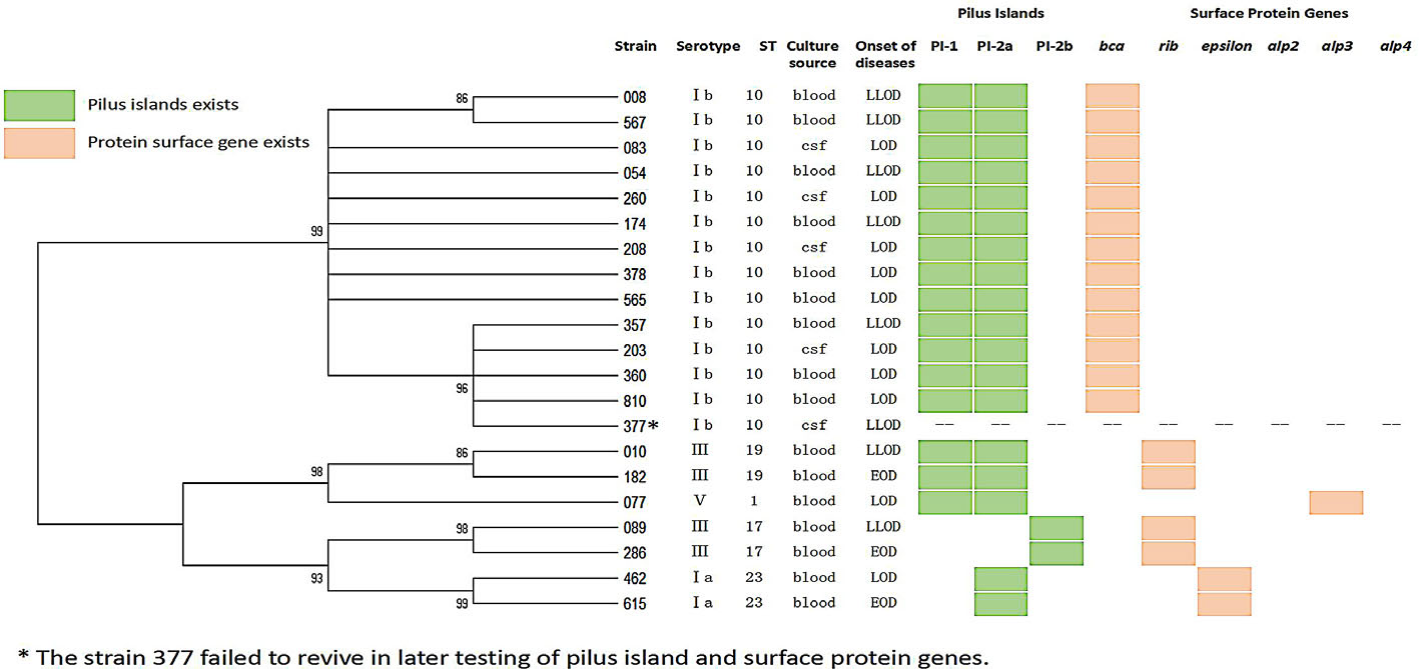
Dendrogram constructed from the multi-locus sequence typing (MLST) profiles of seven housekeeping genes of 21 GBS isolates.

Over three years, a total of 21 invasive GBS diseases have been reported, including 16 isolates recovered from blood and five from cerebrospinal fluid (**Figure 1**).

### High Prevalence of Type Ib/ST10 Strains Among Infants’ Invasive GBS Disease

The most widespread in the population, 85% of the isolates, were serotypes Ib (14/21, 66.7 percentiles, P < 0.05) and III (4/21, 19.0%). A total of five STs that were clustered into five CCs were identified (**Table 2** and **Figure 1**). ST10 was the most common type of sequence (14/21, 66.7%, P < 0.05), followed by ST23 (2/21, 9.5%), ST19 (2/21, 9.5%), and ST17 (2/21, 9.5%). All serotype Ib GBS isolates belonged to CC10 ST10.

**Table 2:** Relationships between sequence type and serotype in 21 invasive GBS isolates.

Clonal complex (CC)	Sequence type (ST)	Serotype				
		Ia(n/%)	Ib(n/%)	III (n/%)	V (n/%)	Total
CC23	ST23	2 (9.5)	–	–	–	2 (9.5)
CC19	ST19	–	–	2 (9.5)	–	2 (9.5)
CC17	ST17	–	–	2 (9.5)	–	2 (9.5)
CC10	ST10	–	14 (66.7)^a^	–	–	14 (66.7)^a^
CC1	ST1	–	–	–	1 (4.8)	1 (4.8)
Total		2 (9.5)	14 (66.7)^a^	4 (19.0)	1 (4.8)	21 (100.0)

a Number of Ib/ST10 strains vs all other isolates.

### Clinical Manifestations in Type Ib/ST10 GBS Strain Infection

These invasive GBS diseases are summarized in clinical presentations in **Table 3**.

**Table 3:** Demographics and clinical features of infants with type Ib/ST10 GBS invasive infections compared with those with other GBS invasive infections.

Clinical characteristics	Type Ib/ST10 strains (n =14)	Other type strains (n = 7)	Total (n =21)
Preterm (gestational age <37 weeks)	4 (28.6)	0 (0)	4 (19.0)
Low birth weight(<2,500 g)	2 (14.3)	0 (0)	2 (9.5)
Gender			
Male n (%)	9 (64.3)	4 (57.1)	13 (61.9)
Female n (%)	5 (35.7)	3 (42.9)	8 (38.9)
Onset of diseases			
EOD	0 (0)	3 (42.9)	3 (14.3)
LOD	8 (57.1)^a^	2 (28.6)	10 (47.6)
LLOD	6 (42.9)^a^	2 (28.6)	8 (38.1)
Bacterial infections			
Sepsis	8 (57.1)	5 (71.4)	13 (61.9)
Meningitis	9 (64.3)^a^	2 (28.6)	11 (52.4)
Pneumonia	8 (57.1)	5 (71.4)	13 (61.9)
Cellulitis	0 (0)	0 (0)	0 (0)
Clinical complications			
Septic shock	2 (14.3)	0 (0)	2 (9.5)
Respiratory failure	1 (7.1)	0 (0)	1 (4.8)
Heart failure	2 (14.3)	0 (0)	2 (9.5)
Multi-organ failure	0 (0)	0 (0)	0 (0)
Discharge outcome			
Recovered	12 (85.7)	5 (71.4)	17 (81.0)
Transferred to other hospitals	1 (7.1)	0 (0)	1 (4.8)
Died	0 (0)	0 (0)	0 (0)
Abnormal neurology at discharge	1 (7.1)	0 (0)	1 (4.8)
Discharge requested	0 (0)	2 (28.6)	2 (9.5)

All data were expressed as number (percentage %). ^a^Infants with type Ib/ST10 GBS diseases vs all other isolates.

Most of these infants were male (61.9%), and term-born [gestational age (GA) >37 weeks, 17/21, 81.0%], and only one (4.8%) at GA <30 weeks. There were a higher number of LOD cases (10/21, 47.6%) than LLOD (8/21, 38.1%) and EOD cases (3/21, 14.3%). The Ib/ST10 type was significantly predominant in LOD and LLOD (14/18, 77.8%, p < 0.05) in comparison with the distribution of serotypes and genotypes in LOD and EOD. In addition, type Ib/ST10 GBS strains occupy more than four-fifths share of infantile meningitis (9/11, 81.8%, p < 0.05). Infants with type Ib/ST10 GBS disease had a considerably higher rate of meningitis (9/14, 64.3%, p < 0.05) and clinical complications (5/14, 35.7%, p < 0.05), including septic shock (n = 2), respiratory failure (n = 1), and heart failure (n = 1) when compared with other types.

### Surface Protein Gene Profile and Pili

In genes encoding *α*-C protein and *α*-like proteins (Alps), we found discrepancies between isolates (**Figure 1**): ST1 isolates possessed gene *alp3*, encoding Alp3. The ST10 strains had gene *bca*, encoding *α*-C protein, the ST19, and ST17 isolates possessed the gene encoding Rib protein, and the ST23 strains had the gene encoding the epsilon protein. The PI of GBS had three types: PI-2a, PI-2b, and PI-1 + PI-2a. All ST17 were PI-2b, all ST23 were PI-2a. All ST10, ST19, and ST1 were PI-1 + PI-2a.

### Multiple Resistance of Invasive GBS Isolates

**Figure 2:**
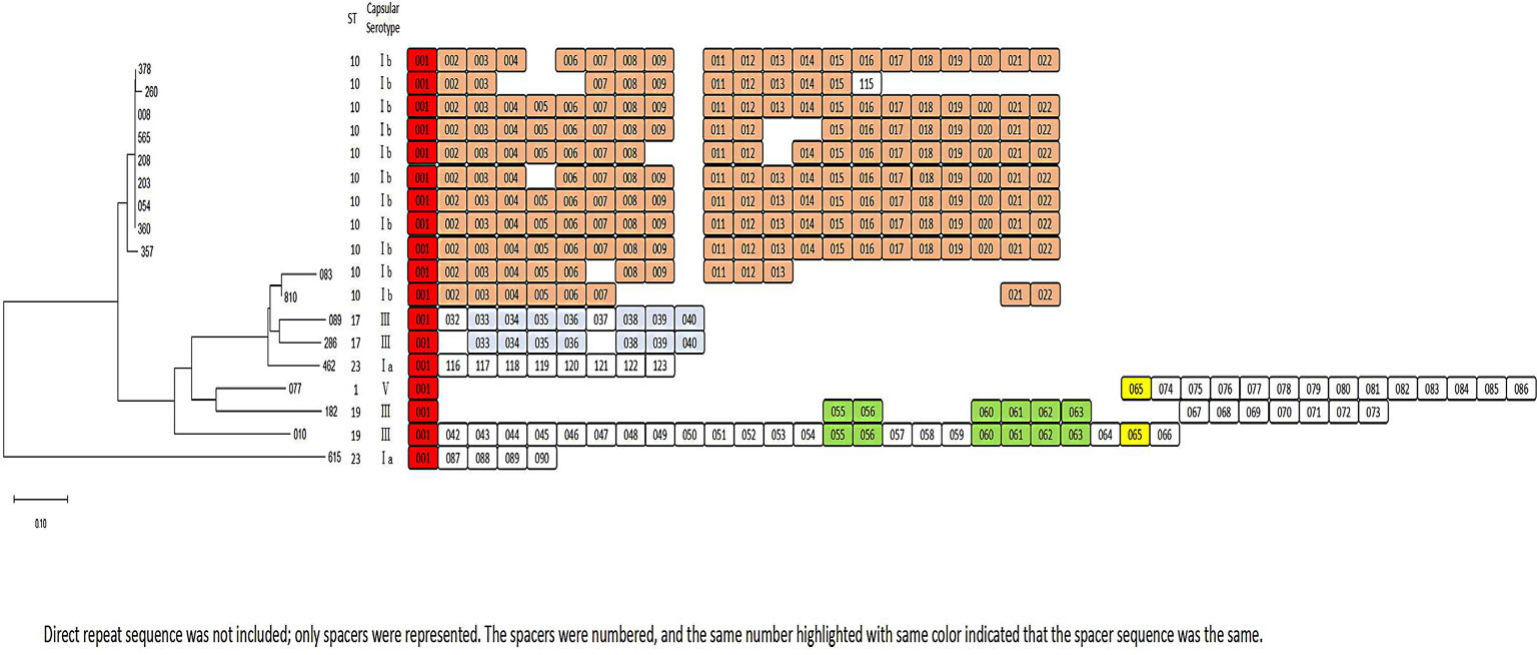
Dendrogram constructed from the CRISPR1 profiles of GBS strains.

**Figure 2** indicates that there is a link between serotypes and resistance-virulence. All isolates were susceptible to penicillin G, vancomycin, cefepime, ceftriaxone, and cefotaxime. Resistance to erythromycin was found in 19 (90.5%) of invasive isolates, similar to resistance to clindamycin, resistance to tetracycline in seven (33.3%), and resistance to levofloxacin in 17 (81.0%). Type Ib/ST10 strains have noted significantly higher levels (100%) of erythromycin, clindamycin, and levofloxacin resistance as we observed antimicrobial resistance profiles by serotype and by ST. In contrast, none of the Ib/ST10 strains were resistant to tetracycline. Approximately 90.5% (19/21) of the invasive GBS isolates were multi-resistant; all Ib/ST10 types showed resistance to erythromycin, lincomycin, and FQ. Type III/ST17 is resistant to tetracycline, erythromycin, and lincomycin, and V/ST1 and III/ST19 are resistant to tetracycline, erythromycin, FQ, and lincomycin.

### Antimicrobial Resistance Genes Present in GBS Strains

All lincosamide- and macrolide-resistant strains, including all Ib/ST10 strains (**Figure 2**), possessed gene *ermB* and ST19 isolates also include genes *mefA* and *lnuB*. The tetM and tetO genes have been linked with tetracycline resistance, and they were carried by all invasive GBS isolates except for type Ib/ST10 strains. All FQ-resistant isolates (Ib/ST10, III/ST19, and V/ST1) had amino acid changes within the quinolone resistance-determining region (QRDR) of *gyrA* and *parC* compared with the reference sequence. The mutations were S79F (15/17) or S79Y (2/17) in the product of *parC* and S81L in *gyrA* (17/17).

### CRISPR Analysis and Phylogenetic Reconstruction

**Figure 3:**
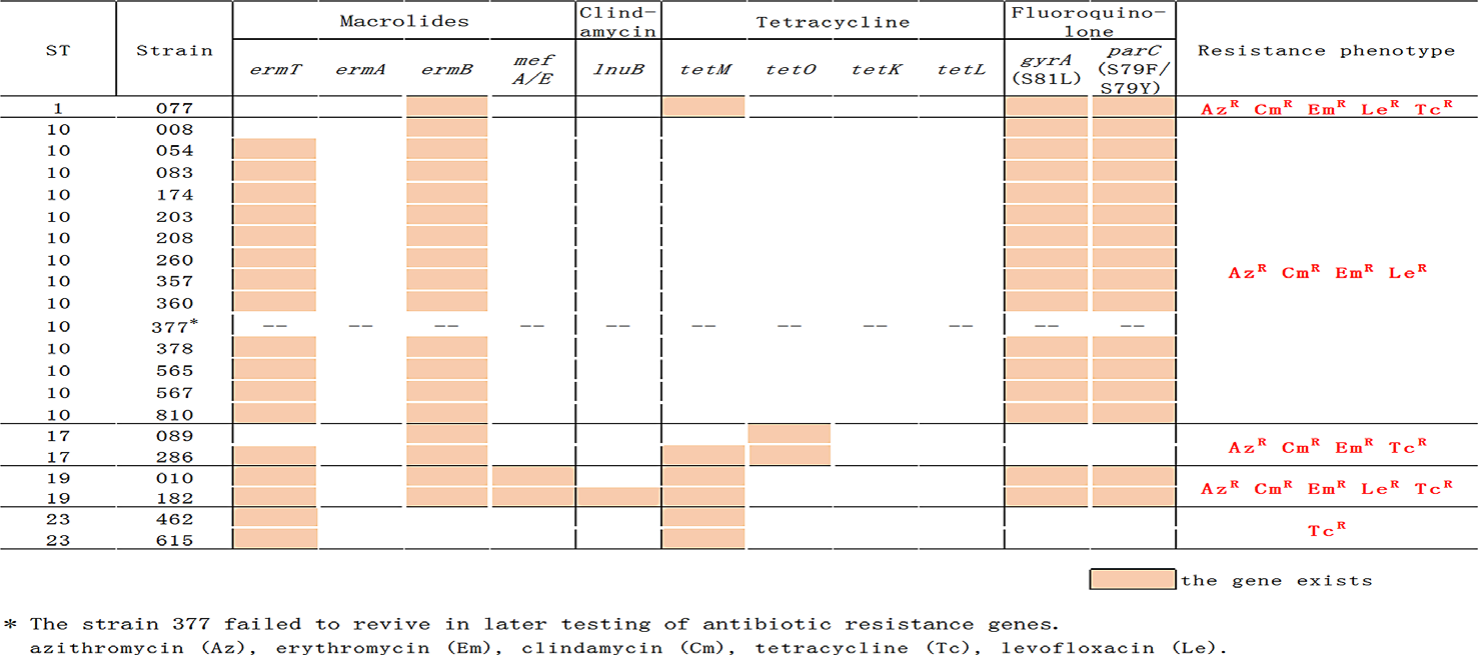
Distribution of antibiotic resistance gene and phenotypes in the GBS isolates.

A CRISPR1 research was carried out to further study the genetic ties between invasive GBS isolates. As shown in **Figure 3**, except for three strains, 377 failed to revive in later testing of CRISPR1; 492, and 678 had sequence gaps at CRISPR1. All the other 18 strains had intact CRISPR1 sequence structures. The CRISPR sequences of ST10 strains mostly contained No. 02–022 spacers, compared with that of other strains.

## Discussion

Numerous research studies on GBS molecular epidemiology and genomic diversity have been carried out over the past few decades (Lawn et al., 2017; Li et al., 2017; Madrid et al., 2017; Furfaro et al., 2018). However, data relating to the genetic features of the prevalent circulating lineages and in particular geographical areas remain scarce. Here, we characterized a collection of 14 invasive type Ib/ST10 GBS strains isolated from infants with invasive GBS disease in Shanxi, China during 2017–2020.

Serotype Ib GBS infections are more prevalent in Asia than in other regions of the world (Lawn et al., 2017; Li et al., 2017; Madrid et al., 2017; Furfaro et al., 2018). In Japan, 19.8% of pregnant women and 34.9% of asymptomatic neonates from 2006 to 2009 were confirmed to be Ib GBS serotypes (Wakimoto et al., 2011). In South Korea, the GBS serotype Ib accounted for 22% of infections in adults (Uh et al., 2005). In Taiwan, Lin et al. found that serotype Ib GBS accounted for 21.6% of the isolates collected from a mixed population of pregnant women, neonates (Lin et al., 2014), and non-pregnant adults, indicating the commonality of the serotype. Similarly, in China, Ji et al. (2019) reported that the serotype Ib strains were the second most common cause of invasive infant disease (28.7%) from 2015 to 2017, particularly in the Northern and Northeastern regions. The 66.7% serotype Ib GBS prevalence in this study indicates that the diffusion of serotype Ib in China is a significant pathogen. To our knowledge, this is the highest level of infant invasive Ib GBS serotype disease ever recorded.

Previously, we have recorded that a high incidence of ST10 strains among both infants and colonized pregnant women (Zhang et al., 2020) strongly correlated with serotype Ib. The GBS MLST method was developed by Jones and colleagues using seven housekeeping genes (adhP, atr, glcK, glnA, pheS, sdhA, and tkt) (Jones et al., 2003). Associations between sequence type (ST) and serotype have been previously reported (Lawn et al., 2017; Li et al., 2017; Madrid et al., 2017; Furfaro et al., 2018), with varying levels of correlation. Observed trends reveal variation between serotype Ib and II within ST10 (Kang et al., 2017; Lo et al., 2019), with serotype Ib mostly associated with CC10, including ST8, ST10, and ST12. While it has been suggested that there could be a horizontal transfer of capsular genes in the GBS population (Luan et al., 2005), we observed that all serotype Ib strains belonged to CC10 ST10.

CRISPRs, the bacterial adaptive immune defense mechanism used to defend against foreign nucleic acids, are a family of non-contiguous DNA repeats interspersed by unique spacer sequences of constant length (Gholizadeh et al., 2017; Koonin and Makarova, 2019; Westra et al., 2019). The phylogenetic relations between the subspecies can be studied using CRISPR analyzes (Sorek et al., 2008; Hidalgo-Cantabrana et al., 2018). The presence of two GBS CRISPR systems: CRISPR1 and CRIPR2 has been recorded, in which CRISPR1 was active due to its high diversity of repeat arrays (Lopez-Sanchez et al., 2012). Furthermore, CRISPR1 sequences in GBS represent a new and effective method to accurate population genomics research for this species and to gain a better understanding of the dissemination of specific clones. In our study, we found that the CRISPR1 sequences of type Ib/ST10 GBS strains were highly conserved, indicating the limited genetic diversity of the strains.

We also reported the clinical manifestations of type Ib/ST10 invasive GBS strains in infants. For the majority of LOD and LLOD cases in our community, Type Ib/ST10 GBS was the most dominant invasive clone. The adaptation of unique GBS clonal complexes to a particular niche(s) was not without precedent. The CC17 lineage of GBS (mostly composed of clonal serotype III strains) is widely recognized to have been adapted to the neonatal niche, particularly late-onset neonatal disease (Musser et al., 1989). Additionally, more than 90% (210/229) of the invasive serotype V isolates were also closely associated with ST1 clone isolates between 1992 and 2013 (Flores et al., 2015). Furthermore, another expansion of a single clone causing human disease, ST283, has been identified in Southeast Asia (Barkham et al., 2019). Here, we notice that type Ib/ST10 clonal strains are also expanding rapidly among infant niches, particularly in the late onset neonatal invasive infection niche. Serotype Ib GBS strains are associated with more severe clinical presentations (Ji et al., 2019; Kao et al., 2019), including higher rates of septic shock, disseminated intravascular coagulopathy, and multi-organ failure, leading to higher mortality. We found that out of the 52.4% (11/21) of our patients with positive CSF cultures or clinical meningitis, type Ib/ST10 accounted for 81.8% (9/11, P < 0.05). Moreover, infants with type Ib/ST10 GBS diseases had significantly higher rates of clinical complications (5/14, 35.7%, p < 0.05), including septic shock, respiratory failure, and heart failure compared with others. Thus, type Ib/ST10 GBS diseases were indeed associated with high illness severity.

Penicillin is the medicine preferred for the prevention and treatment of GBS disease, with macrolides such as erythromycin and lincosamides, such as clindamycin, recommended for patients with a *ß*-lactam allergy (Li et al., 2017; Furfaro et al., 2018; Hayes et al., 2020). Although several studies revealed high macrolide resistance rates across serotype Ib GBS strains globally (Guo D. et al., 2018; Hayes et al., 2020), we found that the erythromycin resistance rate of type Ib/ST10 (100%) strains was higher than reported. In GBS, FQ resistance emerged in the early 2000s and continues to grow around the world, with rates reaching up to 40% in Asian countries (Wehbeh et al., 2005; Wang et al., 2013; Hays et al., 2016; Wu et al., 2017; Hayes et al., 2020). FQ resistance is predominantly associated with CC-10 and CC-19 isolates, which are conferred by point mutations in the QRDR, mostly at codon position 81 in gyrA and 79 or 83 in parC. We observed that 100% type Ib/ST10 strains were resistant isolates and had amino acid changes within the QRDR of gyrA and parC compared with the reference sequence. Thus, all Ib/ST10 GBS isolates in our study had multi-drug co-resistance to erythromycin, clindamycin, and levofloxacin. FQ resistance was of limited clinical relevance here, given that FQ is contraindicated in neonates and their expectant mothers who form the largest patient groups. However, one cannot exclude the possibility of selection, fixation, and worldwide dissemination of highly epidemic multi-drug-resistant GBS clones through the extensive use of FQ, as the extensive use of tetracycline has specifically selected GBS clones infecting humans in the 1970s (Da Cunha et al., 2014; Hays et al., 2016).

Most GBS strains isolated from humans are reported to be resistant to tetracycline, and in particular, the acquisition of resistant elements, tetO, and tetM, by a subset of GBS clones, has led to their selection and expansion (Da Cunha et al., 2014; Hayes et al., 2020). Expansion of this GBS clone has led to the emergence of strains more adapted to colonizing and infecting humans. Particularly, this has led to the dissemination of the CC17 hypervirulent clone, which is associated with invasive neonatal infections. However, in our study, it is noteworthy that all type Ib/ST10 strains analyzed were sensitive to tetracycline. To investigate the tetracycline resistance rate of type Ib/ST10 GBS strains in public literature, we detailed the documented cases worldwide (**Table 4**) (Kang et al., 2017; Martins et al., 2017; Wu et al., 2017; Guo et al., 2018; Simoni et al., 2018; Wu et al., 2019). Among the 14 GBS strains in the literature, except for one, Ib/ST10/eps/PI-1 + PI-2a strain, that possessed the *tetM* gene, all others were sensitive to tetracycline. Serotype Ib is typically defined by CC10/bca/PI-1 + PI-2a (Jones et al., 2003). In our study, all type Ib/ST10 isolates possessed the same characteristic protein (bca/PI-1 + PI-2a) and tetracycline sensitivity, consistent with existing literature. Thus, at least for Ib/ST10 GBS strains, tetracycline resistance is unlikely to be the driving force for clone emergence. We cannot disregard that clonal expansion may also be driven by other factors, for example, herd immunity or enhanced virulence of the clones that had undergone recombination. Studies elucidating the cause of driven clonal expansion or contributions to GBS host–pathogen interactions should be conducted (Flores et al., 2015; Teatero et al., 2015).

**Table 4:** Information on the susceptibility to tetracycline of type Ib/ST10 GBS strains obtained through the literature search.

Country (study period)	Sample source	No. of isolates(n/%)	Surface protein genes	Pilus island	Tetracycline susceptibility	Resistance Genes	Ref./isolate/ID^a^
South Korea (1995–2015)	Invasive infant infections	3/3.1	none		S		Kang et al., 2017
China (2002–2012)	Invasive or colonizing infections	2/2.3			S		Wu et al., 2017
Portugal (2005–2015)	Invasive or colonizing infection	1/0.5	eps	PI-1,PI-2a		tetM	Martins et al., 2017
China (2008–2015)	Invasive infant infection and colonizing adult infections	3/3.0	hylB,lmb,scpB,bac,Alpha-C		S		Wu et al., 2019
Italy (2010–2016)	Invasive and colonizing adult infections	4/36.4	Alpha-C		S		Simoni et al., 2018
China (2015–2018)	Invasive or colonizing infections	3/12.5	Alpha-C		S		Guo D. et al., 2018

a Ref, Reference.

Our study has several limitations. Firstly, the study was conducted at a single center and the number of strains collected was relatively small. Additionally, most of our invasive GBS strains were type Ib/ST10, with other types having smaller case numbers. Further research enrolling multiple medical centers to obtain a more representative view of the Ib/ST10 GBS strain type is warranted. Secondly, we only employed PCR to study the microbiological characteristics of the GBS strains, as traditional targets lack the level of definition concerning genetic relatedness and pathogenic features. Going forward, a large-scale DNA sequencing approach to elucidate the population genetics of type Ib/ST10 GBS strains should be used.

In conclusion, we are the first to report the clinical and microbiological characteristics of type Ib/ST10 GBS strains isolated from infants with invasive GBS disease in China. Considering the probable clonal expansion and the emerging multi-drug resistant isolates, ongoing monitoring for type Ib/ST10 GBS infections is warranted. Moreover, further studies focused on understanding novel adaptive traits and more aggressive treatments are of importance in the future.

## Data Availability Statement

The original contributions presented in the study are included in the article/supplementary material. Further inquiries can be directed to the corresponding authors.

## Ethics Statement 

The study protocol was approved by the ethics committee of Shanxi Children’s Hospital Shanxi Maternal and Child Health Hospital. This study required neither study-specific nor any other interventions and the data were analyzed anonymously. Therefore, written informed consent from the enrolled patients was waived by the ethics committee.

## Author Contributions

Conception and design: LZha, LM, and W-JK. Acquisition of data (laboratory or clinical): LZha, W-JK, LZhu, and X-HZ. Data analysis and interpretation: L-JX, CG, and Q-HL. Drafting of manuscript and/or critical revision: LZha. All authors contributed to the article and approved the submitted version.

## Funding

This work was supported by the youth research fund from the Health Commission of Shanxi, China [No.2017087] and Shanxi Children’s Hospital Shanxi Maternal and Child Health Hospital, China [No.202018] and [No.202033].

## Conflict of Interest

The authors declare that the research was conducted in the absence of any commercial or financial relationships that could be construed as a potential conflict of interest.

## Appendix—Search Approach

We searched PubMed databases for English-language articles, using the following keywords/phrasing: “Streptococcus agalactiae” OR “streptococcus group B” OR “group B streptococcal” AND “ST10” OR “Ib”. Only the different collection years were selected for repeated reports with the same author or agency. Susceptibility to tetracycline of type Ib/ST10 strains were detected in six reports (**Table 4**).
